# Conformable electrochemical devices for closed-loop wound management

**DOI:** 10.3389/fbioe.2023.1331567

**Published:** 2023-11-22

**Authors:** Jie Li, Zhangping Li, Jian Xiao, Chenyao Nie

**Affiliations:** ^1^ School of Pharmaceutical Sciences, Cixi Biomedical Research Institute, Wenzhou Medical University, Wenzhou, Zhejiang, China; ^2^ The Quzhou Affiliated Hospital of Wenzhou Medical University, Quzhou People’s Hospital, Quzhou, Zhejiang, China

**Keywords:** wound management, conformable device, closed-loop, electrochemical biosensing, on-demand treatment

## Abstract

Chronic wounds arising from accidents, surgeries, or diseases impose a significant clinical and economic burden, underscoring the need for effective solutions to prevent severe complications. Recent advancements in materials science and electrochemical technology have facilitated the development of conformable electrochemical platforms for detection and management, incorporating monitoring, diagnosis, and treatment. Nevertheless, current wound detection and therapy systems face challenges related to the stability and specificity of sensor monitoring, as well as the need for on-site and comprehensive evaluation criteria to offer timely treatment guidance and follow-up care. This review provides a comprehensive overview of the closed-loop management system, emphasizing wound biomarker detection, wound assessment, and on-demand treatment, ultimately culminating in an integrated wound management approach by conformable electrochemical devices. Additionally, we explore the challenges, opportunities, and future prospects of soft and stretchable electrochemical biosensors, with the aim of enhancing the efficiency and timeliness of wound management.

## 1 Introduction

Wounds resulting from accidents, surgeries, or chronic conditions pose a significant clinical challenge and impose a substantial economic burden on global healthcare systems. Effective wound management is not only about alleviating physical discomfort but also stands as a crucial healthcare necessity. Neglected or inadequately treated wounds have the potential to escalate into severe medical issues, including infections, sepsis, amputations, and, at times, even fatalities (Sen, 2021). According to a survey conducted by the World Health Organization, the issue of wounds has affected more than 305 million people around the world. It is estimated that by 2024, global expenditures related to wound care will reach $80 billion (Yao et al., 2022). Traditional wound treatment, often involving dressing removal or sample examination, is time-consuming, expensive, and uncomfortable. A patient-friendly tool is needed to monitor disease progression, guide treatment, and reduce costs (Romanelli et al., 2013).

Recent advancements in materials fabrication, sensing techniques, and device integration strategies have opened up numerous opportunities in various healthcare management systems (Mathew et al., 2020; Sun et al., 2021). In particular, conformable electrochemical devices stand out due to their flexibility, breathability, biocompatibility, and their capability to continuously analyze biomarkers in personalized wound management (Mirvakili and Langer, 2021; Tang et al., 2021). Electrochemical biosensors are cost-effective, portable, and seamlessly integration into electronic acquisition modules, making them promising for comprehensive evaluation of the wound progress and treatment in the intricate wound microenvironment. Furthermore, to enhance patient outcomes and achieve greater efficiency and precision in wound management, electrochemical devices are undergoing continuous evolution. They are shifting from single-function devices that primarily focus on biomarker sensing or treatment to more interconnected wound management systems (Mathew et al., 2020). This evolution is leading to the development of interconnected wound management systems, which seamlessly coordinate various facets of the wound healing process. These systems cover everything from biomarker detection, wound evaluation, and on-demand treatment, ultimately culminating in the creation of a closed-loop wound management system that seamlessly integrates all these modules. This logical progression in technology promises a more holistic and efficient approach to wound care, benefiting both patients and healthcare providers (Mathew et al., 2020; Mirvakili and Langer, 2021; Tang et al., 2021; Yang et al., 2023).

**FIGURE 1:**
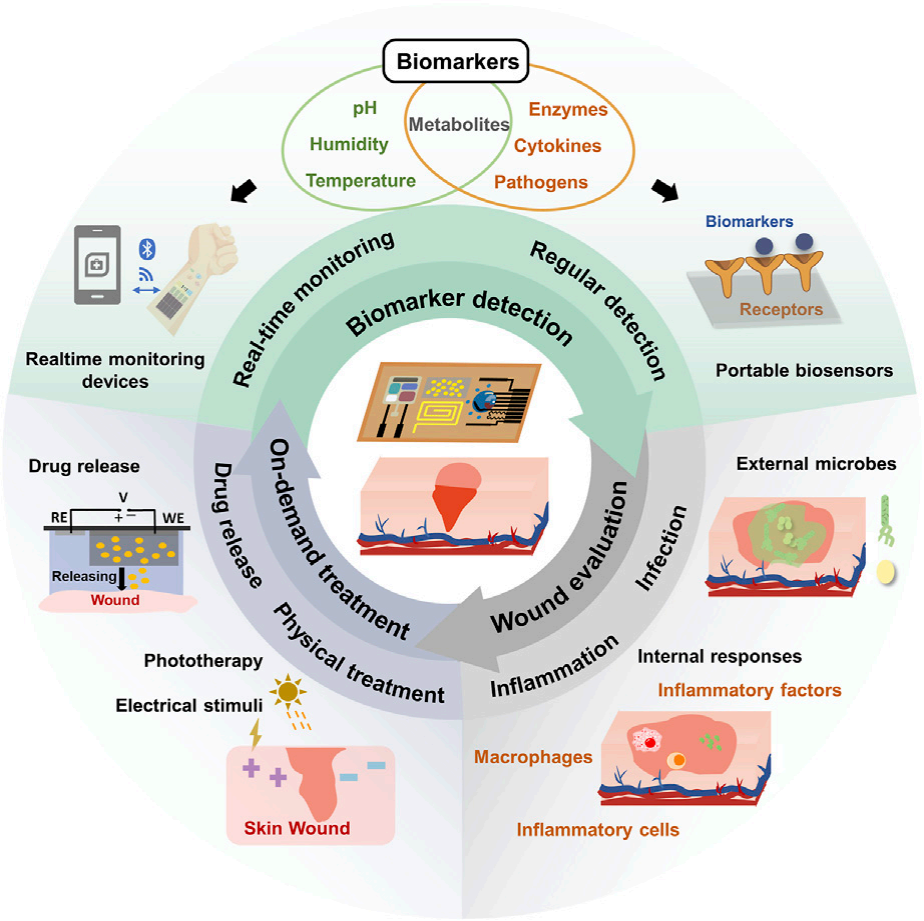
Schematic illustration of conformable electrochemical device for closed-loop wound management.

In this review, we provide an overview of recent advancements in closed-loop wound management systems utilizing conformable electrochemical devices. As illustrated in Figure 1, we begin by introducing the wound biomarkers and the associated electrochemical detection techniques. These techniques are designed to collect essential information through real-time monitoring or periodic detection, tailored to the unique characteristics of different biomarkers. Next, a comprehensive wound condition evaluation criteria and strategies were introduced. Furthermore, we explore electrochemical-based wound treatment approaches, encompassing physical treatments and diverse drug delivery strategies. Our primary aim is to provide an extensive and in-depth perspective on the use of cutting-edge adaptable electrochemical devices in the field of wound management. This review advocates for an integrated closed-loop wound management platform that combines biomarker detection, holistic evaluation, and on-demand treatment, ultimately creating an adaptive and efficient approach to wound care.

## 2 Biomarkers detection

Wound environments encompass a multitude of measurable indicators crucial for comprehensive wound assessment and treatment guidance. These biomarkers provide valuable insights into the wound’s condition and the healing process (Tang et al., 2021). To address this complexity, we provide a concise overview of current electrochemical detection techniques, categorizing them into two modes: real-time monitoring and regular assessment. Real-time monitoring is applied to holistic wound indicators like temperature, pH, and humidity, and small molecule metabolites (such as uric acid, nitric oxide, etc.), while regular detection is employed for biomarkers recognized by bioreceptors that can be detected by disposable devices, such as metabolites, cytokines, and enzymes. This classification allows for a targeted and tailored strategy in wound management, ensuring a cohesive approach to assessing and addressing wound conditions.

### 2.1 Brief introduction to electrochemical detection techniques

Electrochemical detection is a valuable tool for precisely converting wound markers into electrical signals in complex wound environments, ensuring high sensitivity and specificity. Electrochemical sensors stand out for their cost-effectiveness, user-friendliness, and portability compared to alternative methods, making them an ideal choice for *in vitro* diagnosis (Li et al., 2021). These sensors employ various techniques, including amperometric, voltammetric, potentiometric, and impedance methods. In amperometric methods, analyte concentration directly correlates with the measured current. Enzyme sensors, such as uricase-modified sensors for uric acid detection, are commonly used. Voltammetry, the most prevalent technique, captures redox currents in relation to analyte concentration. Potentiometric sensors detect potential shifts resulting from ion concentration changes, while impedance spectroscopy quantifies analytes based on their impedance response (Piro and Reisberg, 2017; Liu et al., 2021; Pollard et al., 2021). Wound related biomarkers and corresponding electrochemical detection methods are summarized in Table 1.

**TABLE 1:** Summary of wound related biomarkers and corresponding electrochemical detection methods.

	Biomarkers	Method	Sensing elements	Detection range	Data transfer	Reference
Biomarkers for real-time detection	pH	Potentiometry	PEDOT	4–8	Bluetooth	Shirzaei Sani et al. (2023)
		Potentiometry	PANI	5–9	Bluetooth	Yang et al. (2022)
		Potentiometry	PANI	3–9	NFC	Xu et al. (2021)
		Voltammetry	PEDOT:PSS	4–10	Bluetooth	Liu et al. (2021)
		Potentiometry	PANI	4–9	Bluetooth	Gao et al. (2021)
	Humidity	Impedance	PEDOT: PSS		NFC	Tessarolo et al. (2021)
		Capacitance	IFRep gel		Bluetooth	Yang et al. (2022)
	Uric acid	Voltammetry	UOx	0–300 μM	Bluetooth	Pal et al. (2018), RoyChoudhury et al. (2018)
		Voltammetry	rGO/AuNPs	100–800 μM	NFC	Xu et al. (2021)
		Voltammetry	chitosan/PB	0–1000 μM	Bluetooth	Liu et al. (2021)
		Voltammetry	PB	0–100 μM	Bluetooth	Shirzaei Sani et al. (2023)
		Voltammetry	PA/CNT	100–1000 μM	None	Jarošová et al. (2019), Simoska et al. (2020)
		Voltammetry	CUAs	100–700 μM	None	Simoska et al. (2020)
	NO	Voltammetry	CUAs	1–100 μM	None	Simoska et al. (2020)
	PYO	Voltammetry	CUAs	1–250 μM	None	Simoska et al. (2020)
		Voltammetry	PA/CNT	1–118 μM	None	Jarošová et al. (2019)
		Voltammetry	MXene/AuNPs/peptides	1–100 μM	NFC	Shi et al. (2023)
Biomarkers for regular detection	Cytokines (TNF-α, TGF-β1, IL-6, IL-8)	Voltammetry	MB	TNF-α:0–2 ng/mL; TGF-β1: 0–150 pg/mL; IL-6: 0–30 ng/mL; IL-8: 0–30 ng/mL	Bluetooth	Gao et al. (2021)
	Cathepsin, Deoxyribonuclease	Capacitance	IFRep gel	0–500 ng/mL	NFC	Yang et al. (2022)
	Sortase A	Voltammetry	MXene/AuNPs/peptides	1 pg/mL to 100 ng/mL	NFC	Shi et al. (2023)

PEDOT- poly(3,4-ethylenedioxythiophene); PANI- polyaniline; PEDOT: PSS- poly(3,4-ethylenedioxythiophene): Poly (styrenesulfonate); IFRep gel-Inflammation-responsive gel (IFRep gel); UOx- urate oxidase; PB- prussian blue; PA/CNT- Polyacrylamide-coated, carbon nanotube; CUAs- carbon ultramicroelectrode arrays; MB- methylene blue.

Electrochemical sensors can provide a versatile and efficient approach to monitor wound biomarkers through real-time monitoring as well as regular detection modes. These methods significantly contribute to enhancing wound management by offering precise insights into wound conditions and facilitating well-informed treatment decisions.

### 2.2 Biomarkers for real-time detection

Wound management is a multifaceted process involving various factors such as temperature, pH, and moisture levels, which serve as holistic biomarkers for real-time sensing. It's important to note that most holistic biomarkers are monitored in conjunction with other biomarkers to provide a comprehensive view of wound conditions from various perspectives. Additionally, small molecule metabolites, closely associated with inflammatory and infectious processes, can be continuously monitored as well. These metabolites are usually detected by using enzyme-based biosensors employing amperometric methods (e.g., glucose) or voltammetric methods.

**FIGURE 2:**
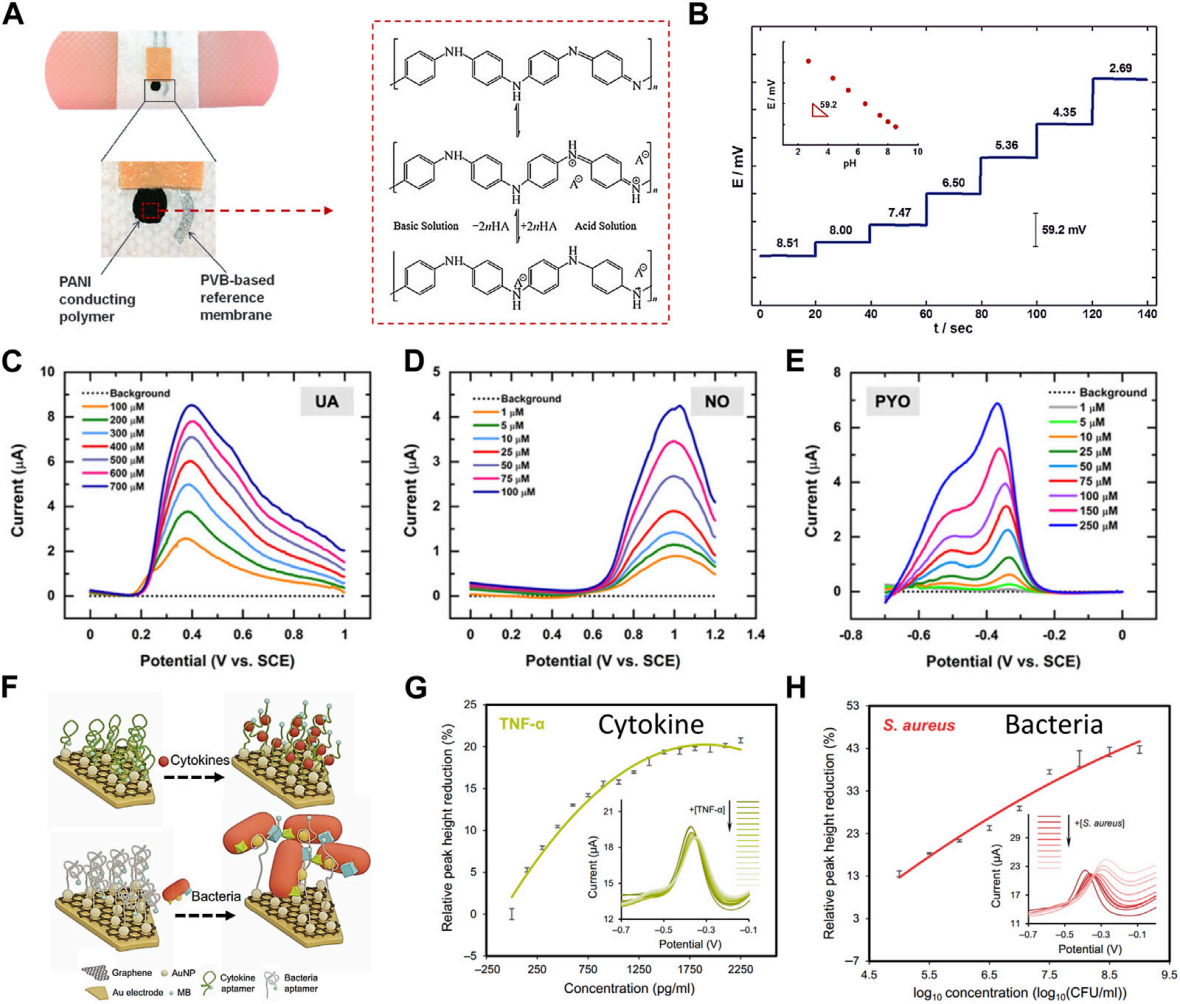
Real-time monitoring **(A–E)** and regular detection **(F–H)** of electrochemical devices for various wound biomarkers. **(A)** The images depict a printed potentiometric sensor on an adhesive bandage, and illustration of the polyaniline protonation-deprotonation pathway from base conditions (top structure) to acid conditions (bottom structure) (Cascioferro et al., 2014). (Copyright 2014 *Electroanalysis*) **(B)** The potentiometric time-trace of a pH bandage sensor ranges from pH 8.51 to 2.69, with an inset showing the EMF dependence versus pH for the PANI conducting polymer ISE (Jayaseelan et al., 2014). (Copyright 2014 *Electroanalysis*) Additionally, **(C–E)** display square wave voltammetric current responses (background subtracted) for several biologically relevant concentrations of **(C)** PYO, **(D)** NO, and **(E)** UA in simulated wound exudates (Simoska et al., 2020). (Copyright 2020 *ACS Sensors*) **(F–H)** Aptamer-based sensors for regular detection of cytokines and bacteria, **(F)** presents a schematic of the sensing mechanisms, and **(G–H)** show variations in relative peak height based on voltammetric spectra of the TNF-α and *S. aureus* sensors (Gao et al., 2021). (Copyright 2021 *Science Advances*).

When the integrity of skin is destroyed, many factors will lead to the change of temperature in and around the wound. It is found that the temperature is the highest in the unhealed and infected wounds, and it decreases with the healing of the wounds (Power et al., 2017; Gethin et al., 2021). Lou and colleagues, by monitoring the temperature of the wound in real time, gets the specific situation of the wound (Lou et al., 2020). For instance, a sudden temperature increase or prolonged high temperature may indicate infection or delayed healing (Gianino et al., 2018; Prasad Mahindrakar et al., 2023). Wound pH is another critical element in the wound healing equation, significantly influencing essential processes like collagen formation, inflammation, angiogenesis, and tissue oxygen utilization (Romanelli et al., 2013; Pang et al., 2023; Derwin et al., 2023). Among the pH sensing materials, polyaniline (PANI) is reported as a type of widely used conductive polymer that can sensing pH change through the protonation-deprotonation pathway (Figure 2A). And, because of their reversible property of protonation-deprotonation reaction, PANI based sensors can continuously monitoring pH value in a wide pH range (Figure 2B). In the wound environment, moisture levels are equally vital. An adequately humid setting can promote tissue growth and collagen proliferation (Weller, 2009; Kruse et al., 2015), while facilitating the autolysis of necrotic tissue (Nuutila and Eriksson, 2021). However, excessive exudate may not necessarily be beneficial for wound healing, as it can hinder the process (Zhang et al., 2021; Zhang et al., 2022). Striking the right moisture balance is critical because both excessive and insufficient moisture can impede wound healing (Okan et al., 2007). Notably, pH is often monitored by potentiometry and Voltammetric technology, and humidity is often monitored by impendence and capacity.

The wound-environment interface contains electroactive small molecule metabolites, such as glucose, uric acid, and nitric oxide, closely linked to inflammation and infection. These metabolites significantly affect wound healing. Detecting and monitoring these metabolites offers valuable insights into wound conditions and informs appropriate treatments. For example, high glucose levels can harm blood vessels, impair nerve function, reduce circulation, and weaken the immune system, all vital for wound healing. Prolonged uncontrolled blood sugar can result in chronic wounds, which are more susceptible to infection due to impaired healing (Zhu et al., 2019). Uric acid (UA) serves as a critical indicator of wound severity, with its concentration increasing as wounds become more severe. Excess uric acid levels can hinder healing by promoting inflammation, especially in chronically hypoxic wounds (Fernandez et al., 2012; Fernandez et al., 2014; Faruk Hossain and Slaughter, 2021). Nitric oxide (NO) has anti-inflammatory and antibacterial properties, regulates angiogenesis, and influences collagen deposition, playing a crucial role in various cell activities during wound healing. Wounds in the inflammatory stage and those infected by bacteria produce significant NO amounts (Schwentker et al., 2002; Schwentker and Billiar, 2003; Luo and Chen, 2005; Cinelli et al., 2019; Wu et al., 2021; Li et al., 2022). Bacterial metabolites with redox activity such as pyocyanin (PYO) serve as an infection indicator, which is also applied as biomarker for real-time detection. As shown in Figures 2C–E, UA, NO, and PYO with redox activity are able to be simultaneously detected in wound samples or simulators using a flexible carbon microelectrode array (CUAs) sensor. Square wave voltammetry (SWV) was employed to evaluate the sensor’s response, determining the limits of detection (LODs) and linear dynamic range (LDR) in simulated wound fluid. It was found that the characteristic SWV current response of PYO and uric acid increased with the increase of analyte concentration in the micromolar range determined by *in vitro* and *in vivo* concentrations (Simoska et al., 2020). As Figures 2F–H shows, a multiplex immunosensor can realize the in-situ analysis of wound microenvironment, inflammation and infection status through real-time quantitative evaluation of pro-inflammatory factor TNF-α and bacterial load. The concentration of TNF-α in wound fluid of unhealed ulcer is higher than that of healed ulcer. *Staphylococcus aureus* is a dominant species in all types of chronic wound samples. The peak current height of each adaptive sensor decreases with the increase of target concentration, in which the monitoring range of TNF-α is 0–2 ng/mL, and the range of *Staphylococcus aureus* is 0–1 × 10^9^ colony forming unit (CFU)/ml, and it has been proved that it has good selectivity (Gao et al., 2021).

### 2.3 Biomarkers for regular detection

In contrast to the holistic biomarkers and some small molecule metabolites, there are specific biomarkers present in the wound interfaces that are related to both external microbes and internal physiological conditions and inflammatory responses. However, the recognition of these biomarkers is not reversible, and they can only be detected with disposable biosensors. Specific detection techniques are necessary to target these biomarkers and provide a precise evaluation of the wound conditions.

Cytokines are small molecular proteins, which are involved in the regulation and coordination of various cell types (Borena et al., 2015) and are very important in cell signal transduction. Its role may be promoting inflammation or inhibiting inflammation (Pilvenyte et al., 2023), strictly control the wound healing process (Efron et al., 2000; Werner and Grose, 2003). Therefore, it is very meaningful to monitor the change of cytokine expression level (Filik and Avan, 2020). Legrand and coworkers reported that tumor necrosis factor-alpha (TNF-α), interleukin-6 (IL-6), interleukin-8 (IL-8) and transforming growth factor-β1 (TGF-β1) are used for wound assessment. Pro-inflammatory cytokines interleukin-1 (IL-1), IL-6 and TNF-α are upregulated in the inflammatory stage of wound healing (Legrand and Martino, 2022), and their concentrations in chronic wounds are higher than those in normal healing wounds (Gianino et al., 2018). TNF-α and IL-1 are necessary in the early stage of wound healing, but the persistent over-expression may lead to the aggravation of tissue damage through the over-activation of immune cells and their protease products (Efron and Moldawer, 2004). TNF-α has pleiotropic, growth regulating and differentiation effects on various cell types. It is a key regulator of inflammatory reaction and plays an important role in inflammation and infection (Pilvenyte et al., 2023). Three days after skin injury, the level of TNF-a in wound fluid reached its peak. IL-1 exists in two forms: IL-1α and IL-1β. IL-1β is a pro-inflammatory cytokine, which can be induced by infections such as *Staphylococcus aureus* and *Pseudomonas aeruginosa*. It can be detected in the wound environment within 24 h after injury, and its concentration reaches its peak within 24–72 h. Infections such as *Staphylococcus aureus* and *Pseudomonas aeruginosa* can induce IL-1β secretion (Efron and Moldawer, 2004; Barrientos et al., 2008; Pilvenyte et al., 2023). TGF-β plays an important role in inflammation, angiogenesis, epithelial re-formation and connective tissue regeneration. With the occurrence of traumatic injury, its expression also increased. For these wound markers, electrochemical device such as aptamer modified.

Matrix metalloproteinases (MMPs) are the main proteases involved in wound healing, among which MMP-9, MMP-2 and tissue inhibitor of matrix metalloproteinases (TIMPs) are the most common components in wound exudates (Power et al.,2017). The contents of MMP-9 and MMP-2 decreased during wound healing (Tarlton et al.,1997). The balance of protease is very important for wound healing. Excessive protease activity will cause damage to extracellular matrix (ECM) and newly formed tissues, thus delaying the healing process. MMP-9 is produced by many kinds of cells and induced by cytokines, growth factors, stress or inflammation (Rayment et al., 2008). TIMPs is a tissue inhibitor of matrix metalloproteinases, which regulates the activity of MMPs (Behm et al., 2012; Lindley et al., 2016). According to the research, the long-term increase of matrix protease level and the decrease of TIMPs level or the abnormal ratio of matrix protease level to TIMPs level are related to wound nonunion, especially the decrease of MMP/TIMP ratio is a good prognostic indicator of wound behavior (Ladwig et al., 2002). Taking advantage of the proteinase activity, MMPs can be detected using substrate peptide-based biosensors. Using MMP-2 as an example, the peptide unit consists of two regions: one for self-assembly and one for bio-recognition. Ion nanochannels can then generate an electrochemical response in response to MMP activities (Wang et al., 2022).

Many pathogenic species, including *Pseudomonas aeruginosa*, *Enterococcus faecalis*, *Escherichia coli* and *Staphylococcus aureus*, are usually distributed in chronic wounds. Therefore, microbiological methods for pathogen identification can be used as qualitative indicators of infection (Simoska et al., 2020). The current research progress, aiming at the detection of bacteria, Enzymes such as lipase, hyaluronidase, antimicrobial peptides, antibacterial peptides, substances on the outer membrane of bacteria (such as lipopolysaccharide (LPS), peptidoglycan and lipopeptide), bacterial flagellin, bacterial DNA, etc., are used to monitor bacteria (Khoshroo et al., 2022; Wang et al.,2020).

## 3 Evaluation of the wound status

Despite the presence of a wide array of strategies and technologies in wearable chemical sensors for wound interfaces and non-invasive biofluids, the parameterization and calibration of these sensor modes have not received enough attention (Ronkainen et al., 2010). It is crucial to emphasize the collection and integration of data from multiplexed and multimodal sensors in the context of wound information. This is imperative for robust post-processing, particularly when considering the physiological (inflammation) and pathogenic (infection) processes at the wound interface (Sempionatto et al., 2022). The comprehensive interpretation of this multiplexed and multimodal data, whether derived from real-time monitoring or regular sensor readings, occupies an important position in the close-loop wound management systems.

### 3.1 Inflammation at the wound interfaces

The body’s immune system plays a crucial role in initiating an inflammatory response as a protective mechanism at the wound interface. This response involves the participation of various signaling molecules, including metabolites, cytokines, and macrophages, which work in concert to regulate the immune response and coordinate the repair process (Raziyeva et al., 2021; Zhao et al., 2016).

**FIGURE 3:**
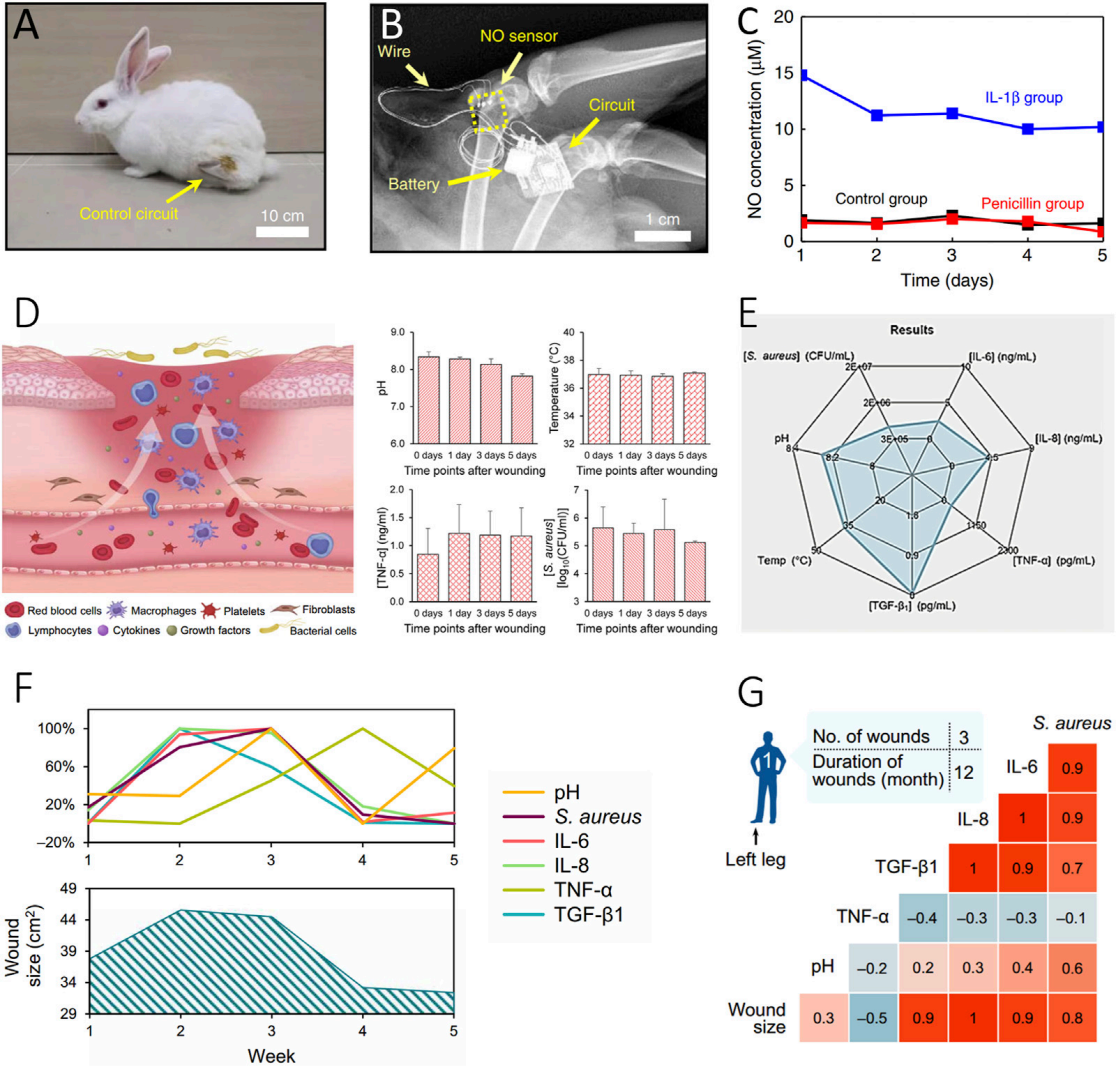
Evaluation of inflammatory status of wound. **(A–C)** Inflammatory Evaluation with a Real-Time NO Monitoring Device in the Puncture Cavity of New Zealand Rabbits (Li et al., 2020). (Copyright 2020 *Nature Communications*) **(A)** X-ray image and **(B)** the implanted NO sensor, wire connections, and wireless module. A photograph of the rabbit after NO sensor implantation with the wireless circuit secured on the thigh. **(C)** High NO concentration correlated with IL-1β-induced inflammation (blue), compared to the untreated (black) and penicillin-treated (red) groups. **(D–G)** Interpretation of Multiplexed Immunosensors with Wound Parameters (Gao et al., 2021). (Copyright 2021 *Science Advances*) **(D)** Illustration of the microenvironment of venous ulcers and *in situ* assessment of pH, temperature, mouse TNF-α, and *S. aureus* using Immunosensors. **(E)** Radar map depicting the integration of different inflammatory-related biomarkers, including TNF-α, IL-6, IL-8, TGF-β1, *S. aureus*, and pH sensors. **(F, G)** Interpretation of sensor-derived data analysis of wound exudate from a patient with a venous ulcer over a 5-week period **(F)** and the resulting correlation matrices **(G)**.

The assessment of inflammation holds paramount importance in effective wound management, with numerous biomarkers intricately involved in this process. NO plays a pivotal role in the regulation of the immune response by influencing key factors such as chemokines, IL-8, TGF-β1, monocytes, and neutrophils, etc., (Luo and Chen, 2005; Cinelli et al., 2019; Wu et al., 2021). In the proliferative stage of wound healing, NO promotes the proliferation of endothelial cells and mediates the production of vascular endothelial growth factor (VEGF) (Luo and Chen, 2005). A study by Li R. et al. illustrated a strong correlation between NO and cytokines, utilizing a real-time wireless NO monitoring platform (Li et al., 2020) (Figures 3A, B). Moreover, NO sensors placed in the puncture cavity of New Zealand rabbits exhibited a robust correlation with the inflammatory cytokine IL-1β and proved adaptable for wound assessment (Figure 3C).

Considering the complex nature of conditions at the wound interface, the need for versatile and integrated in-situ multi-channel inflammatory cytokine detection platforms, along with holistic pH sensors, becomes evident for comprehensive wound condition assessment (Figure 3D). The Lim group’s work showcases the integration of various types of inflammatory data using a flexible multiplexed immunosensor, allowing quantitative interpretation of cytokines like TNF-α, IL-6, IL-8, and the growth factor TGF-β1 through a radar map (Figure 3E) (Gao et al., 2021). This approach also revealed changes in these biomarkers closely correlated with different stages of wound recovery over a period of 5 weeks. The correlation of this inflammatory information with wound conditions offers valuable insights into the healing process (Figures 3F, G). Ultimately, this integrated immune monitoring system serves as a powerful tool for real-time assessment of wound conditions, offering invaluable support for optimizing wound management and making informed treatment decisions.

### 3.2 Infection at the wound interfaces

In the context of chronic wounds, infections present significant challenges as external microorganisms release virulence metabolites. These metabolites cause changes in the overall wound environment and specific biomarker alterations. These alterations hold vital information for early warning and diagnostics, bridging the crucial link between infection and wound assessment to the on-demand treatment.

**FIGURE 4:**
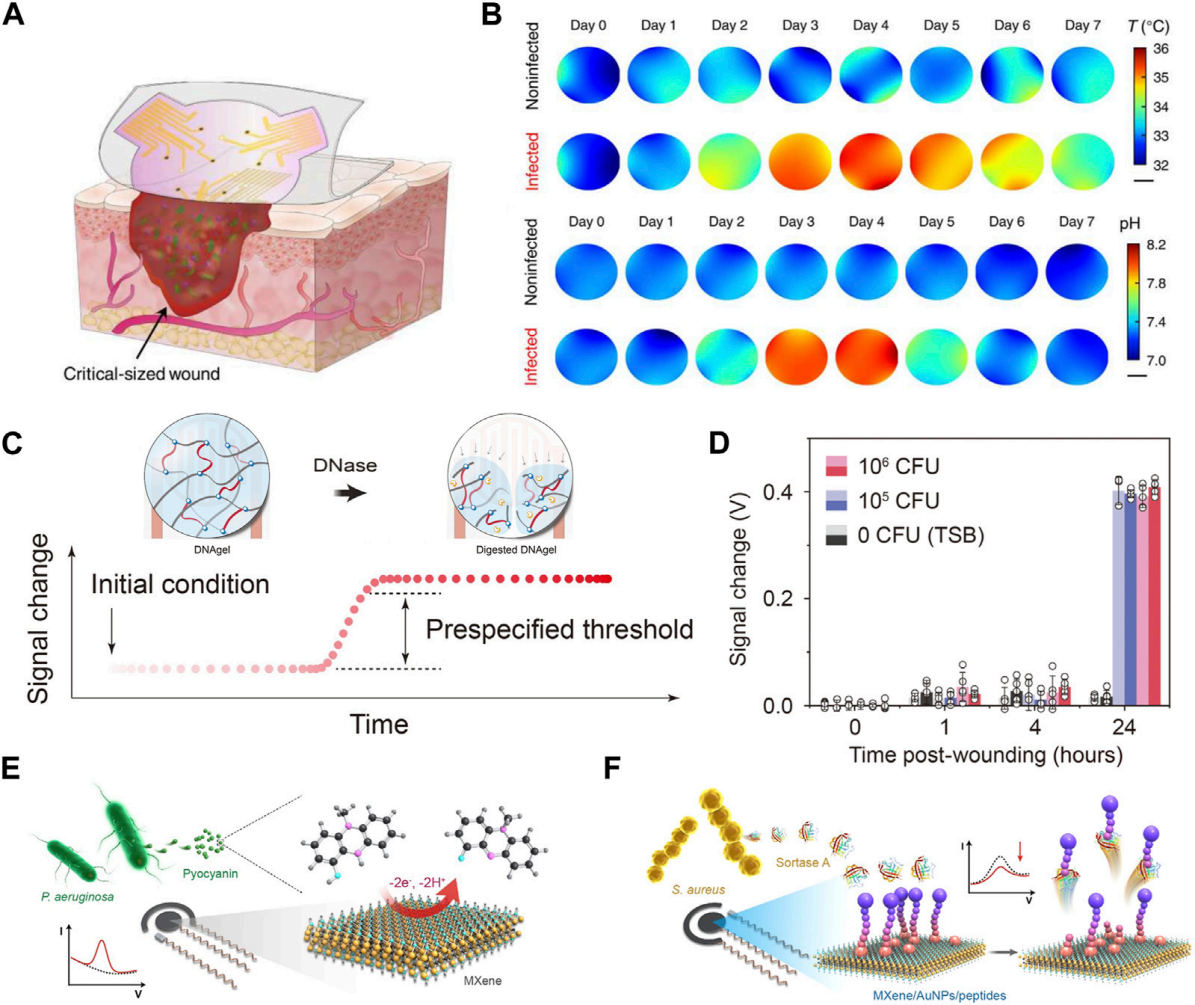
Assessment of wound infection status. **(A)** Illustrates a soft sensor patch equipped with pH and temperature sensor arrays for spatial and temporal monitoring of large and irregular wounds. **(B)** Displays the mapping of daily local pH and temperature (Shirzaei Sani et al., 2023). (Copyright 2023 *Science Advances*) **(C)** Schematics of the infection sensing mechanism for DNAgel-based infectious evaluation. DNAgel degrades upon exposure to DNase, resulting in a change in the capacitance of the sensor. **(D)** Signal changes recorded by a smartphone for *S. aureus* load at the wound site established by wound culture 24 h after wounding (Xiong et al., 2021). (Copyright 2021 *Sci Adv*.) **(E, F)** Schematics of the electrochemical detection of pyocyanin **(E)** based on MXene-modified electrodes and detection of sortase A **(F)** with MXene/AuNPs-modified electrode in the presence of a 100 µM ferrocenylacetic acid solution in PBS solution (Shi et al., 2023).. (Copyright 2023 *Sensors and Actuators B: Chemical*.)

Holistic biomarkers such as temperature, pH, and other vital parameters can be utilized to evaluate infectious status. For temperature, distinct temperature changes were observed in wounds infected with various bacterial strains. Prolonged elevated temperatures may indicate infections, such as those caused by *Staphylococcus aureus*. A Flexible Wound Healing System (FWHS) incorporates a visual indicator and an alarm for automatically detecting abnormal temperature changes exceeding the rectal temperature. This system, tested on both early and late infected wounds, demonstrated varying temperature fluctuations at different stages, confirming its early warning capabilities (Lou et al., 2020). In Gao group, wearable patches with pH and temperature sensor arrays were used to monitor the critical size of full-thickness infected wound defects in diabetic rats (Figure 4A). They observed that the infected wound showed a more uniform pH and temperature in regions 2 and 3 days after infection due to the formation of a uniform biofilm after infection. After treatment on the fourth day, the pH value and temperature value decreased significantly and returned to the level before infection on the seventh day, which indicated that the biofilm was destroyed and finally eliminated after treatment (Figure 4B) (Shirzaei Sani et al., 2023).

The virulence factors associated with conditional pathogens can serve as reliable criteria for assessing infection status (Clatworthy et al., 2007; Mota et al., 2021). For instance, the secretion of deoxyribonuclease (DNase) by bacteria such as *Staphylococcus aureus*, *Pseudomonas aeruginosa*, and *Streptococcus* pyogenes reflects the occurrence of infection (Figure 4C). In this method, a reactive DNAgel with wound extrudes is employed. Elevated levels of DNase trigger the degradation of the DNA hydrogel, resulting in a change in the dielectric constant. This change can be utilized as an indicator to evaluate the infection status. When infection occurs, the bacterial reactive DNAgel contacts with DNase, and the bacterial reactive DNA hydrogel degrades, which leads to the change of dielectric constant in the area above the staggered electrodes, thus triggering the infection alarm on the smart phone. The capability to indicate wound infection *in vivo* was validated with an acute wound model in mice. The widely accepted clinical threshold for laboratory infection diagnosis, ranging from 10^5^ to 10^6^ colony-forming units (CFU), was employed. In the control group, minimal changes were observed. However, in wounds inoculated with live *Staphylococcus aureus* (10^5^ and 10^6^ CFU), a signal change of 0.4 V was detected after 24 h (Xiong et al., 2021) (Figure 4D).

In another study, two typical biomarkers, sortase A and pyocyanin, were employed to indicate infections caused by Gram-positive *Staphylococcus aureus* and Gram-negative *Pseudomonas aeruginosa*. Electrochemical differential pulse voltammetry was utilized for timely evaluation of wound infections and to distinguish between infection types. Results from animal experiments demonstrate the effectiveness of the smart bandage in conducting multi-biomarker analysis of wounds, enabling the early warning of wound infections (Figures 4E, F) (Shi et al., 2023).

The wound evaluation approaches mentioned above are based on specific biomarkers closely related to the status of wound conditions. However, the variation in these wound-related biomarkers is influenced by multiple factors within the complex wound environment. In such cases, artificial intelligence can analyze complex data, offering potential applications in early detection, diagnosis, treatment, prognosis prediction, and prognosis evaluation of wounds, contributing significantly to the field of wound care. Research has successfully integrated artificial intelligence technology into wound management, particularly in the classification of chronic wounds, including diabetic ulcers, lymphatic vessel wounds, surgical wounds, and pressure injuries (Sarp et al., 2021). This integration has facilitated accurate evaluations of wound area and the percentage of granulation tissue (PGT) (Howell et al., 2021), prediction of the healing stage of inflammatory wounds (Kalasin et al., 2022), and overall assessment and management of wounds (Barakat-Johnson et al., 2022). These advancements not only optimize the diagnosis and treatment workflow but also improve the efficiency of the entire wound care process.

## 4 On-demand wound management systems

The progress in the management of wounds has resulted in the emergence of precise, instantaneous, and dynamic on-demand treatment systems that cater to individual requirements (Verdolino et al., 2021; Vivcharenko et al., 2023). Traditional flexible electrochemical devices with singular monitoring functions are no longer adequate. To effectively address the needs of wound management, integrated closed-loop systems that amalgamate monitoring and controllable treatment systems are emerging as a superior option (Pollard et al., 2021).

These integrated closed-loop systems hold immense potential, encompassing diagnosis, early infection warning, treatment effect assessment, and long-term wound management (Liu et al., 2023). Recent years the application of physical treatment (i.g. electrical stimulation), controlled drug release, or combined treatment have been reported in closed-loop wound treatment (Bagherifard et al., 2016; Amjadi et al., 2018; Jiang et al., 2022).

### 4.1 Physical treatment

**FIGURE 5:**
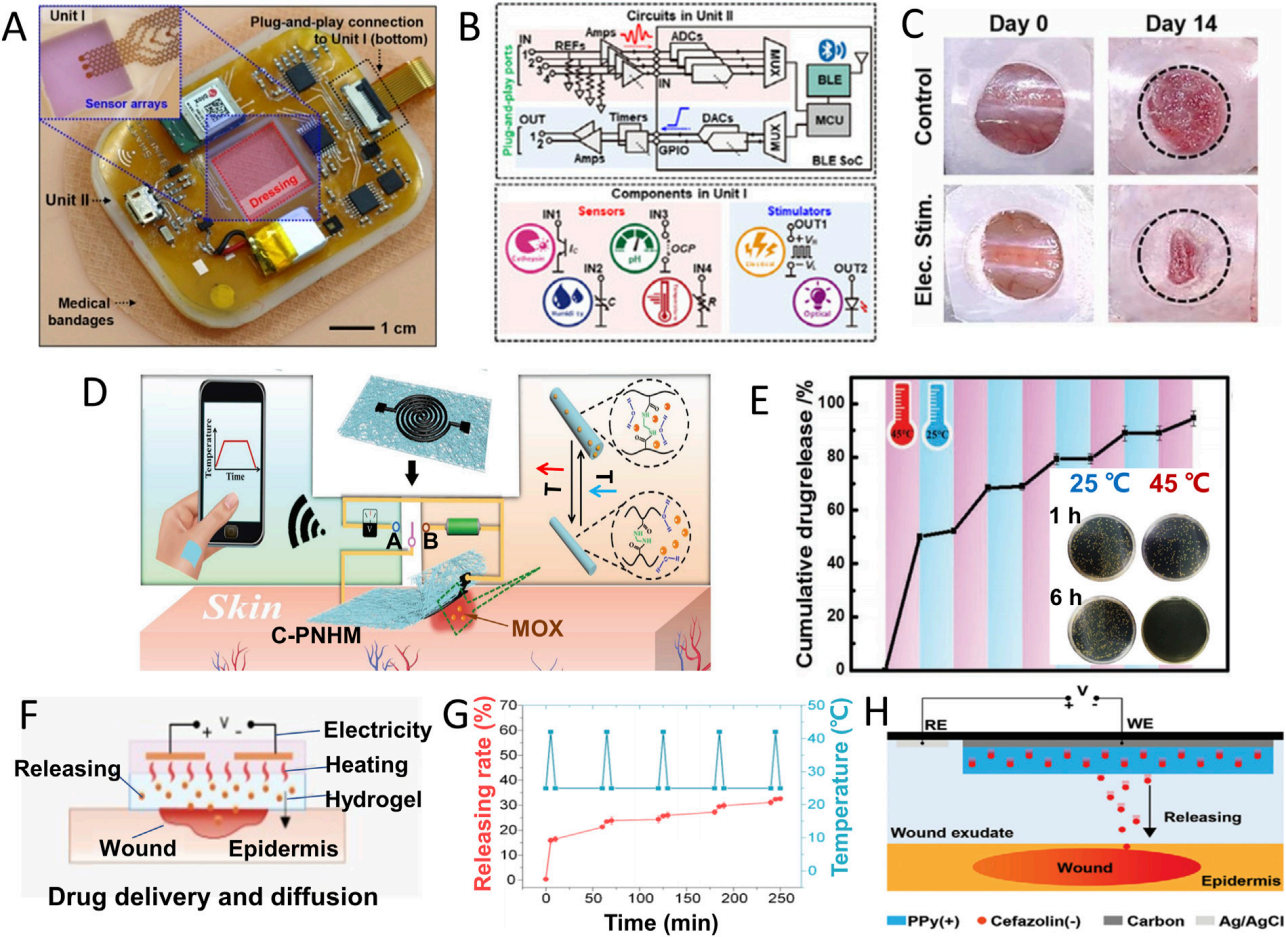
Conformable electrochemical device for wound therapy. **(A)** Photograph of an all-in-one wireless electronic wound dressing system with two units as shown in **(B)** Unit Ⅰ for sensing and optical/electrical stimulations, and Unit Ⅱ for functional control. **(C)** Images of wound closure on day 0 and day 14 without or with electric stimulation by the device in A (Yang et al., 2022). (Copyright 2022 *Nano Today*) **(D)** Schematic illustration of temperature monitoring (double-sided switch connecting to site **(A)** and thermal-induced MOX drug release from C-PNHM (double-sided switch connecting to site **(B)** for on-demand wound management. **(E)** Temperature responsive MOX release from C-PNHM during several temperature change cycle between 25°C and 45°C; inset: viability of bacteria after 1 h or 6 h incubation with C-PNHM fiber at 25°C or 45°C (Gong et al., 2019) (Copyright 2019 *Advanced Functional Material*) **(F)** Schematic diagram of simultaneously exudate removal and drug release in thermal response. **(G)** Cefazolin release under cyclic thermal stimulation (5 min in 42°C and 5 min in 25°C) (Ge et al., 2023). **(H)** Schematic diagram of voltage-controlled drug release in a battery-free wound management system (Xu et al., 2021). (Copyright 2021 *Advanced Functional Material*).

Electrical stimulation (ES) has exhibited its efficacy in regulating cellular proliferation and migration, mitigating inflammation, and expediting wound closure by emulating or amplifying the inherent electromagnetic field’s influence induced by potassium (K^+^) and sodium (Na^+^) ions (Luo et al., 2021). Electrical stimulation application was found to accelerate wound healing and skin remodelling, with increased wound impedance and improved histological characteristics such as skin thickness and the number of skin appendages. Single-cell RNA sequencing (SCRNA-SEQ) analysis confirmed the activation of regeneration-promoting genes in monocytes and macrophages and followed by enhancing tissue regeneration, neovascularization, and skin recovery (Jiang et al., 2022). Yang and coworkers proposed a medic-free wound manage approach by a wireless electrochemical device (Figure 5A) that contains a unit for electrical/optical stimulations (Figure 5B). Figure 5C suggests the electronic dressing devices induced wound healing acceleration successfully with only physical treatment (Yang et al., 2022).

Furthermore, phototherapy, encompassing photodynamic therapy (PDT) and photothermal therapy (PTT), has been demonstrated to accelerate wound healing by impacting macrophage phenotypic metamorphosis, fostering tissue proliferation, and impeding bacterial functionalities, thereby restraining biofilm growth and microbial infections (Xu et al., 2022; Chen et al., 2023).

### 4.2 Controlled drug release treatment

In certain situations, physical treatments alone may not suffice for managing chronic wound conditions, particularly when dealing with infectious microbes. There is a clear need for specific drugs and controlled drug release strategies that can respond actively based on the information and feedback obtained through monitoring and simulation results. This integrated approach offers a more comprehensive and effective way to address the complex and evolving nature of chronic wounds, ensuring that treatment is tailored to individual needs (Luo et al., 2021; Jiang et al., 2022).

One common strategy is thermal-induced drug release, known for its ease of control. Gong et al., integrated a thermally responsive nanomesh film: Screen printing crosslinked poly (N- isopropylacrylamide -N- methacrylamide), with loaded moxifloxacin hydrochloride (MOX) and a dual-functional electrochemical device for wound management. As shown in Figure 5D, this device can monitor temperature changes when double-sided switch connects to site A, and the signal can be wirelessly transmitted to a mobile phone for infection determination. If an increase in temperature was confirmed by infection, turning double-sided switch to site B can active the heater function of device. The heater’s temperature can be precisely controlled by adjusting the applied voltage, ensuring accurate MOX drug release from nanofibers (Figure 5E) for effective bacterial infection control (Gong et al., 2019).

Ge Z and coworkers added an exudate management module to optimize wound management. Self-pumping microfluidic channel, temperature and humidity sensor, liquid metal (LM) heating module and antibiotics-loaded thermosensitive hydrogel are integrated into wearable wound patch (Figure 5F). When the pulse voltage was applied to the LM heating coil, the exudate was removed through microfluidic channel, and the broad-spectrum antibiotic cefazolin was released from the temperature-sensitive hydrogel. The drug release rate at 42°C is significantly higher than that at 25°C, showing controllable and on-demand thermal response release characteristics (Figure 5G). Under the combined action of exudate removal and drug treatment, the wound temperature returned to normal level quickly due to a synergistic effect (Ge et al., 2023). Xu and coworkers adopted conductive polymer and prepared a battery free electronically controlled drug release system offers low power consumption, cost-effectiveness, and rapid response (Figure 5H) (Xu et al., 2021).

Light serves as an optimal stimulus for precise drug delivery owing to its cleanliness, accuracy, and remote controllability. A comprehensive system comprising a temperature sensor, UV-responsive antibacterial hydrogel, and UV-LED enables the monitoring of wound temperature in real-time and the release of antibiotics as needed. Once the presence of infection was confirmed, the UV-LED (365 nm) is applied to activate the release of gentamicin (GS) and the release rate of GS escalates with prolonged exposure to UV irradiation. Upon the release of antibiotic, a gradual decrease in temperature can be observed. Subsequent cycles of UV irradiation maintained this pattern. Animal experiments validated the system’s capability for real-time wound monitoring, infection detection, and effective treatment (Pang et al., 2020).

### 4.3 Combined wound treatment

The combination of electrical stimulation and drugs offers a comprehensive approach to wound management. A multi-channel electrochemical biosensor array is employed to monitor wound biomarkers and regulate drug release via electrodes. The positively charged chondroitin 4-sulfate (CS) hydrogel facilitates the controlled release of dual-function anti-inflammatory and antibacterial peptides (AMP). *In vitro* experiments validated the precise release of AMP and its potent antibacterial activity against diverse pathogens. After treatment, pH and temperature normalized by the fifth and sixth days. The combined treatment group exhibited the highest rate of wound closure, as evidenced by histopathological analysis revealing collagen deposition and the formation of granulation tissue (Shirzaei Sani et al., 2023).

Above all these treatment strategies are effective in different scenarios. Thermal stimulation drug release is common in wound management but comes with drawbacks like high energy consumption and the need for external components. In contrast, electric stimulation release is cost-effective and has low power consumption, although it's limited to charged drugs. Light, as a clean energy source, offers high temporal and spatial resolution and remote control for drug release. The choice of treatment methods is closely tied to specific wound conditions, underscoring the importance of closed-loop wound management (Armstrong et al., 2020; Jiang et al., 2022; Pang et al., 2023).

## 5 Discussion and perspectives

In summary, our review has provided an overview of closed-loop wound management systems based on conformable electrochemical devices. These systems encompass biomarker detection, wound evaluation, and on-demand treatment, offering a comprehensive and efficient approach to wound management. Wound healing is a complex process that demands a thorough understanding of its status. Utilizing various electrochemical techniques to assess multiple wound biomarkers from different perspectives is essential, forming a solid foundation for diagnosis and treatment. Different wound types present distinct microenvironments, requiring adjustments in monitoring parameters for more precise and detailed assessments. The choice of electrochemical strategies depends on the types of biomarkers, ranging from holistic environmental biomarkers requiring continuous monitoring to specific biomarkers necessitating regular detection. The integration of multi-channel electrochemical devices allows for comprehensive monitoring, including wound microflora and their metabolites, by combining various detection modes. This comprehensive evaluation of wound conditions enables the precise identification of infectious or inflammatory states, facilitating timely treatment. Implementing measures to prevent biological contamination of interfaces, enhance sensitivity and specificity, perform active biosensor calibration, reduce response times, lower detection limits, and advance equipment miniaturization, networking, and intelligence is crucial for optimizing electrochemical sensors.

Furthermore, these closed-loop systems can address the challenges posed by multidrug resistance associated with traditional antibiotics. Alternative therapies such as growth factors, NO, metal ions, antimicrobial peptides, and physical modalities like electrical stimulation, light therapy, ultrasound, and heat have emerged as promising options for promoting skin regeneration. Recent developments have seen the application of drug-controlled release, electrical stimulation, photobiological regulation, and other technical methods in closed-loop wound treatment. These advancements underscore the increasing need for innovative design and manufacturing technologies for wearable sensors.

The future of wound management is heading towards intelligent platforms that seamlessly integrate monitoring and treatment, creating a closed-loop for wound detection and treatment. This evolution not only depends on the development of sensing materials but also the incorporation of artificial intelligence algorithms capable of integrating the uncovered bioinformation at the sensor-wound interfaces. With this capability, precise wound models can be reconstructed into comprehensive profiles, enabling much more efficient wound management steps based on personalized situations. A promising approach involves integrating monitoring systems with controllable treatment systems to establish comprehensive closed-loop systems for detection, diagnosis, and treatment.

## References

[B1] Amjadi M. Sheykhansari S. Nelson B. J. Sitti M. (2018). Recent advances in wearable transdermal delivery systems. Adv. Mater 30 (7), 8. 10.1002/adma.201704530 29315905

[B2] Armstrong D. G. Bauer K. Bohn G. Carter M. Snyder R. Serena T. E. (2020). Principles of best diagnostic practice in tissue repair and wound healing: an expert consensus. Diagnostics 11 (1), 50. 10.3390/diagnostics11010050 33396217 PMC7824433

[B3] Bagherifard S. Tamayol A. Mostafalu P. Akbari M. Comotto M. Annabi N. (2016). Dermal patch with integrated flexible heater for on demand drug delivery. Adv. Healthc. Mater 5 (1), 175–184. 10.1002/adhm.201500357 26501166

[B4] Barakat - Johnson M. Jones A. Burger M. Leong T. Frotjold A. Randall S. (2022). Reshaping wound care: evaluation of an artificial intelligence app to improve wound assessment and management amid the COVID-19 pandemic. Int. Wound J. 19 (6), 1561–1577. 10.1111/iwj.13755 35212459 PMC9111327

[B5] Barrientos S. Stojadinovic O. Golinko M. S. Brem H. Tomic-Canic M. (2008). PERSPECTIVE ARTICLE: growth factors and cytokines in wound healing. Wound Repair Regen. 16 (5), 585–601. 10.1111/j.1524-475X.2008.00410.x 19128254

[B6] Behm B. Babilas P. Landthaler and M. Schreml S. (2012). Cytokines, chemokines and growth factors in wound healing. J. Eur. Acad. Dermatol Venereol. 26 (7), 812–820. 10.1111/j.1468-3083.2011.04415.x 22211801

[B7] Borena B. M. Martens A. Broeckx S. Y. Meyer E. Chiers K. Duchateau L. (2015). Regenerative skin wound healing in mammals: state-of-the-art on growth factor and stem cell based treatments. Cell Physiol. Biochem. 36 (1), 1–23. 10.1159/000374049 25924569

[B8] Cascioferro S. Totsika M. Schillaci D. (2014). Sortase A: an ideal target for anti-virulence drug development. Microb. Pathog. 77, 105–112. 10.1016/j.micpath.2014.10.007 25457798

[B9] Chen H. Y. Wu L. J. Wang T. Y. Zhang F. L. Song J. Y. Fu J. (2023). PTT/PDT-induced microbial apoptosis and wound healing depend on immune activation and macrophage phenotype transformation. Acta Biomater. 167, 489–505. 10.1016/j.actbio.2023.06.025 37369265

[B10] Cinelli M. A. Do H. T. Miley G. P. Silverman R. B. (2019). Inducible nitric oxide synthase: regulation, structure, and inhibition. Med. Res. Rev. 40 (1), 158–189. 10.1002/med.21599 31192483 PMC6908786

[B11] Clatworthy A. E. Pierson E. Hung D. T. (2007). Targeting virulence: a new paradigm for antimicrobial therapy. Nat. Chem. Biol. 3 (9), 541–548. 10.1038/nchembio.2007.24 17710100

[B12] Derwin R. Patton D. Strapp H. Moore Z. (2023). Wound pH and temperature as predictors of healing: an observational study. J. Wound Care 32 (5), 302–310. 10.12968/jowc.2023.32.5.302 37094930

[B13] Efron D. T. Most D. Barbul A. (2000). Role of nitric oxide in wound healing. Curr. Opin. Clin. Nutr. Metab. Care 3 (3), 197–204. 10.1097/00075197-200005000-00006 10871235

[B14] Efron P. A. Moldawer L. L. (2004). Cytokines and wound healing: the role of cytokine and anticytokine therapy in the repair response. J. Burn Care Rehabil. 25 (2), 149–160. 10.1097/01.bcr.0000111766.97335.34 15091141

[B15] Faruk Hossain M. Slaughter G. (2021). Flexible electrochemical uric acid and glucosebiosensor. Bioelectrochemistry 141, 6, 107870. 10.1016/j.bioelechem.2021.107870 34118555

[B16] Fernandez M. L. Upton Z. Edwards H. Finlayson K. Shooter G. K. (2012). Elevated uric acid correlates with wound severity. Int. Wound J. 9 (2), 139–149. 10.1111/j.1742-481X.2011.00870.x 21973196 PMC7951012

[B17] Fernandez M. L. Upton Z. Shooter G. K. (2014). Uric acid and xanthine oxidoreductase in wound healing. Curr. Rheumatol. Rep. 16 (2), 396. 10.1007/s11926-013-0396-1 24357442

[B18] Filik H. Avan A. A. (2020). Electrochemical immunosensors for the detection of cytokine tumor necrosis factor alpha: a review. Talanta 211, 17, 120758. 10.1016/j.talanta.2020.120758 32070602

[B19] Gao Y. Nguyen D. Yeo T. Lim S. B. Tan W. X. Madden L. E. (2021). A flexible multiplexed immunosensor for point-of-care *in situ* wound monitoring. Sci. Adv. 7 (21), eabg9614. 10.1126/sciadv.abg9614 34020961 PMC8139589

[B20] Ge Z. Guo W. Tao Y. Sun H. Meng X. Cao L. (2023). Wireless and closed-loop smart dressing for exudate management and on-demand treatment of chronic wounds. Adv. Mater 2023, e2304005. 10.1002/adma.202304005 37547949

[B21] Gethin G. Ivory J. D. Sezgin D. Muller H. O'Connor G. Vellinga A. (2021). What is the "normal" wound bed temperature? A scoping review and new hypothesis. Wound Repair Regen. 29 (5), 843–847. 10.1111/wrr.12930 33987906

[B22] Gianino E. Miller C. Gilmore J. (2018). Smart wound dressings for diabetic chronic wounds. Bioengineering 5 (3), 5030051. 10.3390/bioengineering5030051 PMC6163915 29949930

[B23] Gong M. Wan P. Ma D. Zhong M. Liao M. Ye J. (2019). Flexible breathable nanomesh electronic devices for on-demand therapy. Adv. Funct. Mater 29 (26). 10.1002/adfm.201902127

[B24] Howell R. S. Liu H. H. Khan A. A. Woods J. S. Lin L. J. Saxena M. (2021). Development of a method for clinical evaluation of artificial intelligence–based digital wound assessment tools. JAMA Netw. Open 4 (5), e217234. 10.1001/jamanetworkopen.2021.7234 34009348 PMC8134996

[B25] Jarošová R. McClure S. E. Gajda M. Jović M. Girault H. H. Lesch A. (2019). Inkjet-printed carbon nanotube electrodes for measuring pyocyanin and uric acid in a wound fluid simulant and culture media. Anal. Chem. 91 (14), 8835–8844. 10.1021/acs.analchem.8b05591 31198034

[B26] Jayaseelan S. Ramaswamy D. Dharmaraj S. (2014). Pyocyanin: production, applications, challenges and new insights. World J. Microbiol. Biotechnol. 30 (4), 1159–1168. 10.1007/s11274-013-1552-5 24214679

[B27] Jiang Y. Trotsyuk A. A. Niu S. Henn D. Chen K. Shih C.-C. (2022). Wireless, closed-loop, smart bandage with integrated sensors and stimulators for advanced wound care and accelerated healing. Nat. Biotechnol. 41 (5), 652–662. 10.1038/s41587-022-01528-3 36424488

[B28] Kalasin S. Sangnuang P. Surareungchai W. (2022). Intelligent wearable sensors interconnected with advanced wound dressing bandages for contactless chronic skin monitoring: artificial intelligence for predicting tissue regeneration. Anal. Chem. 94 (18), 6842–6852. 10.1021/acs.analchem.2c00782 35467846

[B29] Khoshroo A. Mavaei M. Rostami M. Valinezhad-Saghezi B. Fattahi A. (2022). Recent advances in electrochemical strategies for bacteria detection. BioImpacts 12 (6), 567–588. 10.34172/bi.2022.23616 36644549 PMC9809139

[B30] Kruse C. R. Nuutila K. Lee C. C. Y. Kiwanuka E. Singh M. Caterson E. J. (2015). The external microenvironment of healing skin wounds. Wound Repair Regen. 23 (4), 456–464. 10.1111/wrr.12303 25857996

[B31] Ladwig G. P. Robson M. C. Liu R. A. N. Kuhn M. A. Muir D. F. Schultz G. S. (2002). Ratios of activated matrix metalloproteinase-9 to tissue inhibitor of matrix metalloproteinase-1 in wound fluids are inversely correlated with healing of pressure ulcers. Wound Repair Regen. 10 (1), 26–37. 10.1046/j.1524-475x.2002.10903.x 11983004

[B32] Legrand J. M. D. Martino M. M. (2022). Growth factor and cytokine delivery systems for wound healing. Cold Spring Harb. Perspect. Biol. 14 (8), a041234. 10.1101/cshperspect.a041234 35667794 PMC9341469

[B33] Li D. Wu C. Tang X. H. Zhang Y. abd Wang T. (2021). Electrochemical sensors applied for *in vitro* diagnosis. Chem. Res. Chin. U 37 (4), 803–822. 10.1007/s40242-021-0387-0

[B34] Li R. Qi H. Ma Y. Deng Y. Liu S. Jie Y. (2020). A flexible and physically transient electrochemical sensor for real-time wireless nitric oxide monitoring. Nat. Commun. 11 (1), 3207. 10.1038/s41467-020-17008-8 32587309 PMC7316789

[B35] Li X. Lu Y. Hu Y. (2022). A wireless and battery-free DNA hydrogel biosensor for wound infection monitoring. Matter 5 (8), 2473–2475. 10.1016/j.matt.2022.06.021

[B36] Lindley L. E. Stojadinovic O. Pastar I. Tomic-Canic M. (2016). Biology and biomarkers for wound healing. Plast. Reconstr. Surg. 138, 18S–28S. 10.1097/PRS.0000000000002682 27556760 PMC4998971

[B37] Liu Y. Li J. Xiao S. Liu Y. Bai M. Gong L. (2023). Revolutionizing precision medicine: exploring wearable sensors for therapeutic drug monitoring and personalized therapy. Biosensors 13 (7), 726. 10.3390/bios13070726 37504123 PMC10377150

[B38] Liu Z. Liu J. Sun T. Zeng D. Yang C. Wang H. (2021). Integrated multiplex sensing bandage for *in situ* monitoring of early infected wounds. ACS Sens. 6 (8), 3112–3124. 10.1021/acssensors.1c01279 34347450

[B39] Lou D. Pang Q. Pei X. Dong S. Li S. Tan W. q. (2020). Flexible wound healing system for pro-regeneration, temperature monitoring and infection early warning. Biosens. Bioelectron. 162, 112275. 10.1016/j.bios.2020.112275 32392156

[B40] Luo J. d. Chen A. F. (2005). Nitric oxide: a newly discovered function on wound healing. Acta Pharmacol. Sin. 26 (3), 259–264. 10.1111/j.1745-7254.2005.00058.x 15715920

[B41] Luo R. Dai J. Zhang J. Li Z. (2021). Accelerated skin wound healing by electrical stimulation. Adv. Healthc. Mater 10 (16), e2100557. 10.1002/adhm.202100557 33945225

[B42] Mathew M. Radhakrishnan S. Vaidyanathan A. Chakraborty B. Rout C. S. (2020). Flexible and wearable electrochemical biosensors based on two-dimensional materials: recent developments. Anal. Bioanal. Chem. 413 (3), 727–762. 10.1007/s00216-020-03002-y 33094369 PMC7581469

[B43] Mirvakili S. M. Langer R. (2021). Wireless on-demand drug delivery. Nat. Electron. 4 (7), 464–477. 10.1038/s41928-021-00614-9

[B44] Mota F. Pereira S. Araújo A. Passos M. Saraiva M. (2021). Biomarkers in the diagnosis of wounds infection: an analytical perspective. TrAC Trends Anal. Chem. 143, 116405. 10.1016/j.trac.2021.116405

[B45] Nuutila K. Eriksson E. (2021). Moist wound healing with commonly available dressings. Adv. Wound Care 10 (12), 685–698. 10.1089/wound.2020.1232 PMC8568799 32870777

[B46] Okan D. Woo K. Ayello E. A. Sibbald G. (2007). The role of moisture balance in wound healing. Adv. Skin. Wound Care 20 (1), 39–53. 10.1097/00129334-200701000-00013 17195786

[B47] Pal A. Goswami D. Cuellar H. E. Castro B. Kuang S. Martinez R. V. (2018). Early detection and monitoring of chronic wounds using low-cost, omniphobic paper-based smart bandages. Biosens. Bioelectron. 117, 696–705. 10.1016/j.bios.2018.06.060 30014943

[B48] Pang Q. Lou D. Li S. Wang G. Qiao B. Dong S. (2020). Smart flexible electronics-integrated wound dressing for real-time monitoring and on- demand treatment of infected wounds. Adv. Sci. 7 (6), 1902673. 10.1002/advs.201902673 PMC7080536 32195091

[B49] Pang Q. Yang F. Jiang Z. Wu K. Hou R. Zhu Y. (2023). Smart wound dressing for advanced wound management: real-time monitoring and on-demand treatment. Mater. &Design 229, 111917. 10.1016/j.matdes.2023.111917

[B50] Pilvenyte G. Ratautaite V. Boguzaite R. Ramanavicius S. Chen C. F. Viter R. (2023). Molecularly imprinted polymer-based electrochemical sensors for the diagnosis of infectious diseases. Biosensors 13 (6), 620. 10.3390/bios13060620 37366985 PMC10296657

[B51] Piro B. Reisberg S. (2017). Recent advances in electrochemical immunosensors. Sensors (Basel) 17 (4), 794. 10.3390/s17040794 28387718 PMC5422067

[B52] Pollard T. D. Ong J. J. Goyanes A. Orlu M. Gaisford S. Elbadawi M. (2021). Electrochemical biosensors: a nexus for precision medicine. Drug Discov. Today 26 (1), 69–79. 10.1016/j.drudis.2020.10.021 33137482

[B53] Power G. Moore Z. O'Connor T. (2017). Measurement of pH, exudate composition and temperature in wound healing: a systematic review. J. Wound Care 26 (7), 381–397. 10.12968/jowc.2017.26.7.381 28704150

[B54] Prasad Mahindrakar B. Goswami A. G. Huda F. Naithani M. Basu S. (2023). Wound pH and surface temperature as a predictive biomarker of healing in diabetic foot ulcers. Int. J. Low. Extrem Wounds 23, 15347346231156962. 10.1177/15347346231156962 37424235

[B55] Rayment E. A. Upton Z. Shooter G. K. (2008). Increased matrix metalloproteinase-9 (MMP-9) activity observed in chronic wound fluid is related to the clinical severity of the ulcer. Br. J. Dermatol 158 (5), 951–961. 10.1111/j.1365-2133.2008.08462.x 18284390

[B56] Raziyeva K. Kim Y. Zharkinbekov Z. Kassymbek K. Jimi S. Saparov A. (2021). Immunology of acute and chronic wound healing. Biomolecules 11 (5), 700. 10.3390/biom11050700 34066746 PMC8150999

[B57] Romanelli M. Miteva M. Romanelli P. Barbanera S. Dini V. (2013). Use of diagnostics in wound management. Curr. Opin. Support Palliat. Care 7 (1), 106–110. 10.1097/SPC.0b013e32835dc0fc 23314016

[B58] Ronkainen N. J. Halsall H. B. Heineman W. R. (2010). Electrochemical biosensors. Chem. Soc. Rev. 39 (5), 1747–1763. 10.1039/b714449k 20419217

[B59] RoyChoudhury S. Umasankar Y. Jaller J. Herskovitz I. Mervis J. Darwin E. (2018). Continuous monitoring of wound healing using a wearable enzymatic uric acid biosensor. J. Electrochem. Soc. 165 (8), B3168–B3175. 10.1149/2.0231808jes

[B60] Sarp S. Kuzlu M. Wilson E. Cali U. Guler O. (2021). The enlightening role of explainable artificial intelligence in chronic wound classification. Electronics 10 (12), 1406. 10.3390/electronics10121406

[B61] Schilrreff P. A. Alexiev U. (2022). Chronic inflammation in non-healing skin wounds and promising natural bioactive compounds treatment. Int. J. Mol. Sci. 23 (9), 4928. 10.3390/ijms23094928 35563319 PMC9104327

[B62] Schwentker A. Billiar T. R. (2003). Nitric oxide and wound repair. Surg. Clin. North Am. 83 (3), 521–530. 10.1016/S0039-6109(02)00207-4 12822723

[B63] Schwentker A. Vodovotz Y. Weller R. Billiar T. R. (2002). Nitric oxide and wound repair: role of cytokines? Nitric Oxide 7 (1), 1–10. 10.1016/s1089-8603(02)00002-2 12175813

[B64] Sempionatto J. R. Lasalde-Ramírez J. A. Mahato K. Wang J. Gao W. (2022). Wearable chemical sensors for biomarker discovery in the omics era. Nat. Rev. Chem. 6 (12), 899–915. 10.1038/s41570-022-00439-w 37117704 PMC9666953

[B65] Sen C. K. (2021). Human wound and its burden: updated 2020 compendium of estimates. Adv. Wound Care 10 (5), 281–292. 10.1089/wound.2021.0026 PMC8024242 33733885

[B66] Shi Z. H. Dai C. B. Deng P. X. Li X. Wu Y. Lv J. J. (2023). Wearable battery-free smart bandage with peptide functionalized biosensors based on MXene for bacterial wound infection detection. Sensors Actuators B Chem. Sens 23, 133598. 10.1016/j.snb.2023.133598

[B67] Shirzaei Sani E. A. Xu C. Wang C. Song Y. Min J. Tu J. (2023). A stretchable wireless wearable bioelectronic system for multiplexed monitoring and combination treatment of infected chronic wounds. Sci. Adv. 9 (12), eadf7388. 10.1126/sciadv.adf7388 36961905 PMC10038347

[B68] Simoska O. Duay J. Stevenson K. J. (2020). Electrochemical detection of multianalyte biomarkers in wound healing efficacy. ACS Sens. 5 (11), 3547–3557. 10.1021/acssensors.0c01697 33175510

[B69] Sun X. Zhang Y. Ma C. Yuan Q. Wang X. Wan H. (2021). A review of recent advances in flexible wearable sensors for wound detection based on optical and electrical sensing. Biosensors 12 (1), 10. 10.3390/bios12010010 35049637 PMC8773881

[B70] Tang N. Zheng Y. Cui D. Haick H. (2021). Multifunctional dressing for wound diagnosis and rehabilitation. Adv. Healthc. Mater 10 (22), e2101292. 10.1002/adhm.202101292 34310078

[B71] Tarlton J. F. Vickery Cj Leaper D. J. Leaper Dj Bailey A. J. Bailey A. J. (1997). Postsurgical wound progression monitored by temporal changes in the expression of matrix metalloproteinase-9. Br. J. Dermatol 137 (4), 506–516. 10.1111/j.1365-2133.1997.tb03779.x 9390324

[B72] Tessarolo M. Possanzini L. Gualandi I. Mariani F. Torchia L. D. Arcangeli D. (2021). Wireless textile moisture sensor for wound care. Front. Phys. 9. 10.3389/fphy.2021.722173

[B73] Verdolino D. V. Thomason H. A. Fotticchia A. Cartmell S. (2021). Wound dressings: curbing inflammation in chronic wound healing. Emerg. Top. Life Sci. 5 (4), 523–537. 10.1042/ETLS20200346 34196717 PMC8589427

[B74] Vivcharenko V. Trzaskowska M. Przekora A. (2023). Wound dressing modifications for accelerated healing of infected wounds. Int. J. Mol. Sci. 24 (8), 7193. 10.3390/ijms24087193 37108356 PMC10139077

[B75] Wang L. Li H. Shi L. Li L. Jia F. Gao T. (2022). *In situ* peptide self-assembly on ionic nanochannel for dynamic monitoring of MMPs in extracellular matrix. Biosens. Bioelectron. 195, 113671. 10.1016/j.bios.2021.113671 34624798

[B76] Wang X. Chen X. Song L. Zhou R. Luan S. A. (2020). An enzyme-responsive and photoactivatable carbon-monoxide releasing molecule for bacterial infection theranostics. J. Mater Chem. B 8 (40), 9325–9334. 10.1039/d0tb01761b 32968746

[B77] Weller C. (2009). “Interactive dressings and their role in moist wound management,” in Advanced textiles for wound care (Berlin, Germany: Springer), 97–113.

[B78] Werner S. Grose R. (2003). Regulation of wound healing by growth factors and cytokines. Physiol. Rev. 83 (3), 835–870. 10.1152/physrev.2003.83.3.835 12843410

[B79] Wu M. Lu Z. Wu K. Nam C. Zhang L. Guo J. (2021). Recent advances in the development of nitric oxide-releasing biomaterials and their application potentials in chronic wound healing. J. Mater Chem. B 9 (35), 7063–7075. 10.1039/d1tb00847a 34109343

[B80] Xiong Z. A. Achavananthadith S. Lian S. Madden L. Ong Z. Chua W. (2021). A wireless and battery-free wound infection sensor based on DNA hydrogel. Sci. Adv. 7 (47), eabj1617. 10.1126/sciadv.abj1617 34797719 PMC8604401

[B81] Xu G. Lu Y. Cheng C. Li X. Xu J. Liu Z. (2021). Battery-free and wireless smart wound dressing for wound infection monitoring and electrically controlled on-demand drug delivery. Adv. Funct. Mat. 31 (26). 10.1002/adfm.202100852

[B82] Xu Y. Chen H. Fang Y. Wu J. (2022). Hydrogel combined with phototherapy in wound healing. Adv. Healthc. Mater 11 (16), e2200494. 10.1002/adhm.202200494 35751637

[B83] Yang C. Yang C. Chen Y. Liu J. Liu Z. Chen H. J. (2023). The trends in wound management: sensing, therapeutic treatment, and “theranostics”. J. Sci-Adv Mater Dev. 8 (4), 100619. 10.1016/j.jsamd.2023.100619

[B84] Yang S. M. Kim H. Ko G.-J. Choe J. C. Lee J. H. Rajaram K. (2022). Soft, wireless electronic dressing system for wound analysis and biophysical therapy. Nano Today 47, 101685. 10.1016/j.nantod.2022.101685

[B85] Yao G. Mo X. Yin C. Lou W. Wang Q. Huang S. (2022). A programmable and skin temperature–activated electromechanical synergistic dressing for effective wound healing. Sci. Adv. 8 (4), eabl8379. 10.1126/sciadv.abl8379 35080981 PMC8791608

[B86] Zhang Y. Lin B. Huang R. Lin Z. Li Y. Li J. (2021). Flexible integrated sensing platform for monitoring wound temperature and predicting infection. Microb. Biotechnol. 14 (4), 1566–1579. 10.1111/1751-7915.13821 33945203 PMC8313280

[B87] Zhang Z. Su R. Han F. Zheng Z. Liu Y. Zhou X. (2022). A soft intelligent dressing with pH and temperature sensors for early detection of wound infection. RSC Adv. 12 (6), 3243–3252. 10.1039/d1ra08375a 35425400 PMC8979260

[B88] Zhao R. Liang H. Clarke E. Jackson C. Xue M. (2016). Inflammation in chronic wounds. Int. J. Mol. Sci. 17 (12), 2085. 10.3390/ijms17122085 27973441 PMC5187885

[B89] Zhu Y. Zhang J. Song J. Yang J. Du Z. Zhao W. (2019). A multifunctional pro-healing zwitterionic hydrogel for simultaneous optical monitoring of pH and glucose in diabetic wound treatment. Adv. Funct. Mat. 30 (6). 10.1002/adfm.201905493

